# Insights on COVID-19 Vaccination in Portugal: A Qualitative Study among Health Professionals and Teachers

**DOI:** 10.3390/vaccines10121984

**Published:** 2022-11-22

**Authors:** Marta Estrela, Catarina Leitão, Tânia Magalhães Silva, Adolfo Figueiras, Fátima Roque, Maria Teresa Herdeiro

**Affiliations:** 1 Department of Medical Sciences, iBiMED—Institute of Biomedicine, University of Aveiro, 3810 Aveiro, Portugal; 2 Faculty of Economics, University of Coimbra, 3000 Coimbra, Portugal; 3 Health Sciences Research Center, University of Beira Interior (CICS-UBI), 6201 Covilha, Portugal; 4 Department of Preventive Medicine and Public Health, University of Santiago de Compostela, 15705 Santiago de Compostela, Spain; 5 Health Research Institute of Santiago de Compostela (IDIS), University of Santiago de Compostela, 15705 Santiago de Compostela, Spain; 6 Consortium for Biomedical Research in Epidemiology and Public Health (CIBER Epidemiology and Public Health—CIBERESP), 15706 Santiago de Compostela, Spain; 7 Research Unit for Inland Development, Guarda Polytechnic Institute (UDI-IPG), 6300 Guarda, Portugal

**Keywords:** vaccine, COVID-19, vaccination hesitancy, perceptions, qualitative, focus group

## Abstract

Background: Vaccination against COVID-19 has had a major impact over the course of the pandemic, leading to a reduced number of hospitalizations and deaths. However, the mass vaccination process has been accompanied by skepticism and hesitancy since its beginning. As health professionals and teachers are important public health actors who can strongly intervene to reduce vaccination hesitancy among their patients and students, respectively, this study aimed to assess their main perceptions towards COVID-19 vaccination. Methods: Two focus group sessions, one with health professionals and the other with teachers, were conducted according to the COREQ checklist. Qualitative data were analyzed through theoretical thematic analysis. Results: In general, none of the groups showed vaccine hesitancy, although both groups had concerns regarding the safety and efficacy of the vaccines. The main concerns of health professionals were mostly related to the long-term impact of the COVID-19 pandemic, while teachers were more worried about the lack of access to reliable information about the COVID-19 vaccination. Conclusions: It is plausible to conclude that it is imperative to provide clear and accurate information for the population in order to avoid vaccination hesitancy.

## 1. Introduction

The Coronavirus Disease 2019 (COVID-19) pandemic has caused successive lockdowns with school and workplace closures [1,2,3]. Almost since its beginning, one of the main global priorities was to create an effective vaccine to contain the impact of the disease and reduce its transmission, especially among those who were most vulnerable. The first vaccine against COVID-19 was initially approved in December 2020, thus kickstarting the vaccination process [4,5]. However, some barriers to widespread vaccination have emerged, such as vaccination hesitancy among the population. Vaccination hesitancy, defined as a “state of indecision and uncertainty about vaccination before a decision” [6], is one of the biggest public health threats identified by the World Health Organization (WHO) [7]. Concerns regarding vaccine safety and effectiveness have been shown to be the main drivers for vaccine hesitancy, as well as trust in competent authorities and communications’ media [8,9,10,11,12]. Considering that populations’ perceptions are among the most important determinants for vaccination hesitancy, it becomes crucial to identify the main insights on COVID-19 vaccination among two of the most influential groups within the general population: health professionals and teachers [13,14,15,16,17,18]. While health professionals can educate and empower their patients by providing correct and up-to-date information on COVID-19 vaccination, teachers also have an essential role in terms of public health education, particularly with their students and even with their parents. 

Moreover, health professionals have been at the forefront of fighting this pandemic, making them a priority for vaccination, particularly due to the risk of infection through their close contact with COVID-19 patients [8]. On the other hand, teachers had to watch the closure of their schools and universities, with strict measures being taken after reopening to contain the spread of the pandemic [9,19].

Thus, as healthcare facilities and classrooms are amongst the most susceptible environments for airborne transmission [20], and since these two groups are included in the priority groups for vaccination in most countries [21], it is important to understand the perceptions of these professionals regarding COVID-19 vaccination, namely the prioritization of different groups within civil society [22]. Unlike quantitative studies, qualitative studies in the form of focus groups allow for the identification of attitudes, knowledge, and expectations that not even the participants are aware of before the focus group sessions and that can arise during the sessions due to social effect. Though there are some cross-sectional studies on the perceptions of these professionals on COVID-19 vaccination [23,24,25], qualitative studies remain scarce, especially at the start of the vaccination process. Therefore, and considering the benefits of complementing quantitative evidence with qualitative results, this study aimed to identify the main perceptions of both these groups on COVID-19 vaccination through the conduct of a focus group study. 

## 2. Materials and Methods

### 2.1. Setting 

This focus group study was conducted in Portugal, which has registered 58.735 physicians, 80.238 nurses, 11.458 dentists, and 16.055 pharmacists [26], as well as 17.064 preschool teachers, 30.986 basic education teachers, 102.077 secondary school teachers, and 36.473 higher education teachers [27,28]. 

### 2.2. Vaccination Plan and Vaccination Status

The vaccination plan started in Portugal on 27 December 2020, and was designed by a special task force dedicated to organizing phases of vaccination, prioritizing the most vulnerable groups—nursing home residents, the elderly, along with health professionals and teachers [22]. Up until the conduct of the focus group sessions, in February 2021, only 3% of the Portuguese population had completed their COVID-19 vaccination plan, and were part of the first priority group [29]. 

### 2.3. Focus Group Conduction

The qualitative study was conducted in February 2021, with participants being recruited through a convenience sample by e-mail, and the sessions being conducted via Zoom, based on the availability of the participants who agreed to participate in the study. To avoid the non-representation of different health professionals and teachers, we aimed to diversify our sample by professional occupations. This study was voluntary, without any kind of incentive, and all the participants gave their explicit written consent to participate in this study and to have the discussion session recorded. The study obtained ethical approval from the Guarda Polytechnic Institute’s Ethics Committee (01/2021). 

Before the beginning of each focus group session, the moderator (F.R.), an experienced researcher in these type of studies [30,31], reminded all participants of the study aims, the recordings that would be performed during the session to facilitate data interpretation, and also that the content matter would always remain confidential, with the data being processed without the participants’ identification. To conduct the focus group sessions, a guide with several open-ended questions and a preliminary content analysis table were developed (see Tables S1 and S2 (Supplementary Materials)), based on the published literature about vaccine hesitancy and COVID-19 [8,32]. The sessions were transcribed verbatim by an independent researcher (C.L.), and then coded by another independent researcher (M.E.). After coding, theoretical thematic analysis was carried out, based on the previously published literature [8,9,33]. The Consolidated Criteria for Reporting Qualitative Research (COREQ) were followed [34].

## 3. Results

Two focus group sessions were conducted via Zoom conference in February 2021. Among the total sixteen participants, thirteen were female and three were male. The health professionals’ group (*n* = 8) was composed of three physicians, three nurses, one pharmacist, and one dentist. The teachers’ group (*n* = 8) was composed of one pre-school teacher, four basic/secondary school teachers, and three higher education professors.

Six themes were identified after transcription and analysis. The identified subthemes can be observed in Table 1 (see quote examples for each subtheme in Table S3):

i. Perceptions on vaccination

Four subthemes were identified regarding vaccination in general: (1) vaccines’ effectiveness and safety; (2) risk perception; (3) access to information; and (4) vaccination hesitancy/acceptance. One of the main motivations pointed out by those who were in favor of vaccination in general was vaccines’ effectiveness and safety, as well as their impact on the eradication of severe diseases. Although some participants considered that taking vaccines should be an individual responsibility to protect the surrounding community, others emphasized that no one should be obliged to obtain a vaccinate. Furthermore, teachers highlighted that access to information regarding the vaccination plan is scarce. Health professionals emphasized that the demand for flu and pneumonia vaccines has increased with the emergence of the COVID-19 pandemic;

ii. Impact of the COVID-19 pandemic

Five subthemes were identified regarding the pandemic’s impact. The long-term consequences of the disease and its possible sequelae, as well as the fear of being infected, have been pointed out by both health professionals and teachers. Per the participants’ feedback, the COVID-19 pandemic has had a major impact within society, especially among those who were the most vulnerable: children and families with low socioeconomic status. Regarding health systems, participants highlighted patients’ clinical conditions deteriorating and the postponement of medical appointments, either through unavailability of resources, or through fear of becoming infected at healthcare facilities, thus leading to inadequate patient support. Health professionals also pointed out that, since they had been at the frontline for over a year, their peers were particularly vulnerable to severe burnout, stress, and other mental health disorders;

iii. COVID-19 vaccination process

Both health professionals and teachers discussed the COVID-19 vaccination process, which had started more than a month before. While concerns regarding lack of vaccines’ availability and poor logistical organization were common between groups, the prioritization of different vulnerable groups was discussed. When the session was conducted, health professionals had already begun to receive their COVID-19 vaccines, allowing for an additional discussion of other groups who were not included at the start of the vaccination process but should have been, such as oncology patients. Among teachers, those having close contact with little children felt that they should be part of a priority group for COVID-19 vaccination. On the other hand, teachers from higher educational levels, especially higher education teachers, felt that they should not be included as a priority group, since their hours of contact with students inside a classroom were clearly much more reduced;

iv. COVID-19 vaccine impact/immunity

Several aspects of COVID-19 immunization were discussed in both sessions. While some participants questioned how effective the immune response would be after vaccination, and even compared it to the acquired immunity after a COVID-19 infection, others were particularly concerned with achieving group immunity through mass vaccination. The risk-to-benefit ratio appeared to be very low among participants: the possible side effects of being vaccinated against COVID-19 were pointed out as unimportant, especially when compared with the expectancy of obtaining immunity against the disease. Furthermore, the possibility of testing the acquired immunity after vaccination and infection through a serological test was also mentioned by some participants. Moreover, considering their concerns regarding both individual and group immunity, the participants from both groups expressed their hope that COVID-19 vaccination might be “the light at the end of the tunnel” to finally end the pandemic;

v. COVID-19 vaccination-related information

Both health professionals and teachers expanded the topic of access to adequate information on COVID-19 vaccination, although teachers explored this topic in much more detail compared to health professionals. While some participants highlighted the lack of information and transparency regarding important topics, such as the research being carried out on COVID-19, which would possibly have a positive impact on vaccine acceptance, others emphasized that a lot of information was updated almost by the minute, thus causing some anxiety and confusion among the population. The topics of fake news, the risk of obtaining information through social media, and of poor news reporting by communications media were also mentioned by both groups. In parallel, the effort made by competent authorities and institutions to disseminate adequate information was brought up, especially by teachers. A major concern highlighted by both health professionals and teachers was the low health literacy of the population, as well as the inaccessible language concerning COVID-19 vaccination;

vi. COVID-19 vaccination hesitancy/acceptance

In contrast to the perceptions regarding vaccination in general, the participants emphasized that being part of a low-risk group was a driver for vaccination refusal, regardless of vaccine effectiveness and safety. However, as there are many vaccines against COVID-19, the possibility of choosing the most “trustworthy” vaccine, as well as the prestige of pharmaceutical companies, appeared to improve the trust for some. However, some interesting phenomena were identified in these sessions, highlighting the skepticism of the participants’ peers towards vaccination. Both the context and the access to reliable information appeared to determine whether people were hesitant to be vaccinated or not. Furthermore, some participants pointed out that a “free riding” phenomenon has been noted among their peers, as they prefer to wait for others to be vaccinated and see whether they respond well or not, and only then decide if they want to be vaccinated or not.

## 4. Discussion

This study assessed the perceptions of health professionals and teachers regarding COVID-19 vaccination before its availability for the general population. To our knowledge, this is the first qualitative study to explore the awareness of these two groups regarding this subject in Portugal. Health professionals were part of the first priority group to receive the vaccine, since they were at the forefront of fighting the COVID-19 pandemic, whereas teachers were not part of that same priority group, despite their significant position, particularly when educating younger people. With this study, it was possible to understand the participants’ main key points of view regarding their perceptions on vaccination, the impact of the COVID-19 pandemic, and the COVID-19 vaccination process.

(i) Perceptions on vaccination

Our findings revealed that both groups recognized the importance of vaccination in general, especially for the prevention of morbidity and mortality, the increase in life expectancy, and the eradication of several diseases, such as the Spanish flu endemic in 1918 [35], smallpox [36], and even type 2 and type 3 wild-type polio viruses [37]. Since the beginning of the COVID-19 pandemic, health professionals have noted that there has been an increase in demand for the influenza and pneumococcal vaccines, which was also observed in other countries [38,39,40]. Moreover, regarding their patients, they had not observed hesitancy in taking any vaccine belonging to the National Vaccination Plan (NVP). The NVP is a free universal program accessible to all people living in Portugal, which was first implemented in 1965, and was last updated in October 2020 [41]. The vaccines included in the program have proven to be effective and safe, providing the greatest health gains. On the other hand, teachers believed that vaccines are safe, necessary for individual protection, and extremely important for the community. However, they considered that there was a lack of information about vaccination in general, and so they needed to undertake their own research, for example, by consulting the national health service website, to know more than what had been made available by health institutions and health professionals. Some teachers also outlined the need to ask their family physicians and pediatricians directly, since they felt that some health professionals assumed vaccination would occur as a scheduled health intervention, but without providing enough data on the subject;

(ii) Impact of the COVID-19 pandemic

Regarding the impact of the COVID-19 pandemic, some of the health professionals’ major concerns included the sequelae and the long-term consequences of the disease, since many of their patients were afraid to come to hospitals and some of the medical specialist facilities were closed to prioritize the treatment of COVID-19 patients, which has led to a significant reduction in medical appointments for over a year. Consequently, there was a decrease in patients’ treatments, which health professionals believed would aggravate other diseases that were previously controlled. Additionally, they have been seeing their patients become more concerned and confused, as they believed they could die from other diseases. Even among health professionals, they described not feeling as productive and well-rested as they should be, that many of them were in a complete state of burnout, and were even thinking of changing their career paths. However, they do not think this would influence their decision to take the vaccine or to recommend it. They highlighted that the specialties at the forefront of the pandemic, such as pulmonology, internal medicine, and infectiology, were the most affected fields of medicine. A cross-sectional study from 2020 [42] analyzed the psychological impact of the COVID-19 outbreak on health professionals and reported that a large percentage had high levels of anxiety, depression, stress, and post-traumatic stress, and at least 30% had moderate to severe levels of emotional exhaustion. The same was observed in other cross-sectional studies in China [43] and Cyprus [44], thus reinforcing the obtained results in our study.

Both health professionals and teachers believed that the COVID-19 pandemic will have a huge impact on society, citing the fact that antidepressants’ sales have significantly increased in young adults and even among those professionals who are combating the disease. Moreover, both groups emphasized the economic and social impact that the COVID-19 pandemic will cause in a high number of families. Since many workplaces were closed during the pandemic, many families saw a sharp reduction in their income, leading to their impoverishment. An anonymous online survey conducted in five different countries showed that Thailand was the most affected country, as it relies heavily on tourism and had to deal with the closure of borders, businesses, and night-time curfews [45]. In Portugal, in which these measures were also applied, the tourism sector is one of the most fundamental activities to generate wealth and employment. Furthermore, young children will also be affected since they had missed basic school skills due to schools’ lockdown. Teachers highlighted that when parents cannot help their children, due to lack of knowledge or availability, they will not be able to help them overcome their difficulties, which can compromise their intellectual development. The majority of scientific data suggest that the COVID-19 pandemic has had a negative influence on student learning, disrupting normal school functioning, and leading to learning losses that have yet to be measured, with the potential to enhance educational inequality [46,47,48]. In a study conducted with 11,158 students belonging to 318 Portuguese schools, negative effects were observed in writing skills, math, and motor-control tasks, especially among less economically advantaged children [49]. On the other hand, the teachers who participated in our study pointed out a positive result that this pandemic may bring: the awareness of respiratory etiquette rules, and the fact that since people have become used to wearing masks, they could become receptive to using masks to protect others when they feel sick;

(iii) COVID-19 vaccination campaign

Concerning the COVID-19 immunization process, health professionals stated that their biggest issues were as follows: (i) lack of available vaccines for the general public, as there were insufficient vaccines even for priority groups in district hospitals with smaller populations; (ii) their patients’ fear of leaving their houses to obtain the vaccine; and (iii) lack of organization of the criteria to select who should receive the vaccine first. Furthermore, while pharmacists were not included in the priority groups, dentists were, but almost no one in that group received it since age was a priority feature. When asked about their patients, health professionals noted that in some hospitals, oncology patients were not prioritized, which could lead to their treatments’ discontinuation owing to infection. Based on compelling evidence that COVID-19 increases morbidity and death in patients with cancer, several professional organizations issued early advice on the role of COVID-19 vaccines in cancer patients, who should definitely be prioritized for vaccination [50,51,52]. On the contrary, patients in palliative care, without any independence, were being immunized, which some health specialists believe to be an inadequate strategy due to their poor immune response. On the other hand, most teachers did not consider themselves to be a priority group, since most of them could be working from their own houses, and because young adults were more willing to follow the rules. However, pre-school and basic school teachers indicated that, since they dealt with young children, they felt they should be part of the priority groups as it was impossible to make students behave properly or to make them follow the rules when using a mask. For health professionals and teachers, the COVID-19 vaccine was seen as the solution for the end of the pandemic, despite the fact that they also believed that some preventative measures might still be applied. While teachers were concerned that vaccination rates would not be enough to reach herd immunity, health professionals considered that there should be research focusing on the immunity given by the vaccine or the infection through serological testing.

Both groups also claimed that there was a lot of misunderstanding regarding COVID-19 vaccination-related information as it was being constantly updated, resulting in a lack of consistency from authorities and institutions, and a generalized feeling of distrust among the general population. Moreover, social communication and social networks were also identified as one of the main factors for disinformation, since they could spread fake news, conspiracy beliefs, or overemphasize negative events while overlooking vaccine benefits. While this might seem unimportant and even expected, health professionals and teachers highlighted that, in Portugal, the health and science literacy of the overall population is poor compared to other countries, which does not allow the general public to utilize critical thinking about the news they might hear or see. Vaccination distrust, due to authorities’ and government disorganization, and fake news released on social platforms such as Facebook and YouTube, were also observed in other studies [53,54]. In teachers’ opinions, this could be easily solved if the scientific community were more transparent in explaining the process of creating a vaccine, and if authorities could adopt a more accessible language for everyone to understand. Both groups claimed that there was a higher distrust in the COVID-19 vaccine compared to other vaccines, due to the speed of the process of creating the COVID-19 vaccines. However, health professionals also emphasized that pharmaceutical companies invested extensive resources into vaccine development, and they could not promote a vaccine that they had not ensured would be safe, since they must maintain their reputation. Both groups also stated that they knew a small percentage of people that did not want to receive the COVID-19 vaccine. Some of the reasons presented included the following: (i) not being included in a risk group, and therefore believing they could be asymptomatic; (ii) working remotely; (iii) wanting to see if there were any side effects in others; (iv) being young and healthy; and (v) the speed of the vaccine creation process, as mentioned before. However, they also highlighted that, due to the context of the COVID-19 pandemic, there would be a lot of people changing their reluctance, since there had been an increase in people dying from the infection.

Our study has, however, some limitations, the most relevant being the reduced sample of both health professionals and teachers. Nevertheless, since these professionals were particularly busy at the time of our study, we were unable to recruit a high number of participants for our study, and schedule a time in which most were available at the same time. Still, our study provides an interesting overview of these professionals’ perceptions regarding the COVID-19 vaccination process right at the start of the vaccination process, which helped identify some dimensions and issues that are not usually identified in cross-sectional studies, although the vaccination rollout process in Portugal has been considered a success when compared with other European countries. Additionally, the results of this type of qualitative study can be of interest to identify items for possible questionnaires to apply in the study population and others with similar characteristics. Furthermore, considering the risk of future pandemics, we believe our study may help with the identification of issues and perceptions at the beginning of future immunization processes, thus helping to avoid the repetition of the same mistakes and ultimately improving these processes.

## 5. Conclusions

This study has revealed the perceptions and concerns of health professionals and teachers regarding the COVID-19 vaccination. Since both groups have an essential role in educating the population, it was crucial to understand their points of view. Both groups emphasized the importance of vaccination in individuals and in the community, both for vaccination in general and for COVID-19 vaccines in particular. While health professionals were more worried with the impact of the pandemic on their patients, and the safety and effectiveness of the vaccine, teachers were more concerned with reaching herd immunity and obtaining trustworthy information from healthcare workers and authorities. In summary, it is plausible to conclude that it is imperative to provide clear and accurate information to the population in order to avoid vaccination hesitancy.

## Figures and Tables

**Table 1:** Identified themes and subthemes.

Perceptions on Vaccination	COVID-19 Vaccine Impact/Immunity
Vaccines’ effectiveness and safety	Individual immunity after vaccination
Risk perception	Group immunity
Access to information	Immunity after infection
Vaccination hesitancy/acceptance	Vaccination effects
	End of the pandemic
**Impact of the COVID-19 pandemic**	**COVID-19 vaccination-related information**
Consequences of the disease	Missing information
Fear	Communications through media and social media
Impact on society	Information update
Impact on health systems (patients)	Institutional information
Impact on health systems (health professionals)	Literacy and language
**COVID-19 vaccination process**	**COVID-19 vaccination hesitancy/acceptance**
Priority groups	Risk perception
Logistics/vaccine availability	Vaccines’ effectiveness and safety
	Trust
	Access to information
	Context

## Data Availability

Not applicable.

## Supplementary Materials

The following are available online at https://www.mdpi.com/article/10.3390/vaccines10121984/s1, Table S1: Guide for focus groups sessions, Table S2: Preliminary content analysis table, Table S3: Thematic analysis’ results with quote examples.
