# Supplementary material for: Insights on COVID-19 Vaccination in Portugal: A Qualitative Study among Health Professionals and Teachers
Source: Vaccines (Basel). 2022 Nov 22;10(12):1984. doi: 10.3390/vaccines10121984 (PMC9781933; doi:10.3390/vaccines10121984)
Supplement: Supplementary file 1 [file vaccines-10-01984-s001.zip › vaccines-2043405-supplementary.pdf]

Table S1 – Guide for focus groups sessions

Kreugers' categories	*Questions*
Opening question	• What are your perceptions about vaccines?
Introductory questions	• Have you been vaccinated against influenza?<br>• If you haven't been vaccinated, what were the motives?
Transition questions	• Do you have any knowledge about vaccination?<br>• Do you think the access to vaccination information is adequate?
Key questions	• Do you think the COVID-19 vaccines are safe?<br>• What are your thoughts on the different COVID-19 vaccines?<br>• Would you take any COVID-19 vaccine? If so, when? If not, why?<br>• Do you think that the information on COVID-19 vaccination has been sufficiently clear? If not, why?<br>• Do you believe that health professionals have the moral obligation of vaccinating against COVID-19?<br>• Considering your professional occupation, do you think you should be prioritized to take the vaccine? Why?<br>• Would you recommend the COVID-19 vaccine to your family and to your patients?<br>• Suppose that you were in charge and could make one change regarding the COVID-19 vaccination. What would you do?
Ending questions	• Of all the things that were discussed, which topics covered do you think are the most important?<br>• Finally, is there anything connected with the pandemic and vaccination, which has not been discussed, that you feel strongly about and would like to bring up now?

Table S2 – Preliminary content analysis table

**1. Perceptions on vaccination**	
Participant	Excerpt
**2. Mention of vaccination experiences**	
Participant	Excerpt
**3. Knowledge on vaccination**	
Participant	Excerpt
**4. Feelings towards COVID-19 vaccines**	
Participant	Excerpt
**5. Perceptions on COVID-19 vaccines information**	
Participant	Excerpt
**6. Thoughts on COVID-19 vaccination strategies (priorities, mandatory vaccination, risk groups, etc)**	
Participant	Excerpt

Table S3 – Thematic analysis' results with quote examples

	Participant	Quote
Perceptions on vaccination		
Vaccines' effectiveness and safety	FG2P1	*"I am 100% in favor of vaccines, and I think it is a fundamental tool, in fact it is well documented [...] at a global level, that several vaccination campaigns were fundamental to improve the health of populations in general, to reduce mortality, increase average life expectancy, etc."*
Risk perception	FG1P7	*"As for the flu vaccination, this year […] there was a great demand."*
Access to information	FG2P1	*"I think the information available is very little, although, in general, people are encouraged to do take the vaccines with very little explanation."*
Vaccination hesitancy/acceptance	FG2P2	*"I think that vaccination should be offered, but I live in freedom and, therefore, it should not be absolutely mandatory."*
	FG2P3	*"[…] this has implications in that community dimension […] and, therefore, the fact that some individuals do not get vaccinated can, in fact, have consequences, and some quite complicated for the population in general."*
Impact of the COVID-19 pandemic		
Consequences of the disease	FG1P4	*"But above all, what worries me the most at this stage are the long-term consequences of patients who had COVID-19, what will this [disease] affect."*
Fear	FG1P8	*"[Cancer] patients who end up contracting the disease, even those considered cured, do not continue the treatments out of fear of the sequelae of the disease."*
Impact on society	FG1P8	*"I think the day after this whole phase has passed is going to be really painful because of what we're experiencing. I think we're going to experience a period of post-traumatic stress that we weren't prepared for and were never prepared for. And people's resilience and resistance are decreasing because the time of the pandemic is prolonged."*
	FG2P2	*"We are going to leave this pandemic in a super degraded socioeconomic situation for a large group of families. From the 8th grade, 9th grade, they are able to do distance learning, they can even be autonomous, but children who are having their first apprenticeships will undoubtedly be very affected. However, if we have the right investment, we can recover them. [...] Now, in relation to families of lower socioeconomic status, we are going to have more and more problems, they were already fragile and will continue to be. They must have a chance to recover. It's just that now there aren't even precarious jobs, there's nothing."*
Impact on health systems (patients)	FG1P7	*"We don't know what consequences this will have, and when we start to see our patients that we've followed for years being decompensated because we didn't give them the due attention, due to all this, we will also feel very guilty [...] this has brought a duality of feelings that is very difficult to manage."*
Impact on health systems (health professionals)	FG1P7	*"And at this moment, I even feel in my colleagues, all of us, a certain shame if we say that we are in burn out. We are all exhausted, tired, but we cannot give up. And what is certain is that when this is over, if it is over, I think we're all going to tilt."*
COVID-19 vaccination process		
Priority groups	FG2P5	*"[...] us [teachers] being a priority here doesn't mean that others are not as well. We are talking about including this group as well. […] we work with a lot of children, small children, and it is impossible to maintain the distance […], and because they are immature, it is normal, they take away the mask, put it down, scratch their nose, put their hand to their mouth, do these things and it's not just to protect ourselves, but also to protect them and to protect those we have at home."*
Logistics/vaccine availability	FG1P1	*"I see with great concern that it [the vaccination] is taking a long time to reach everyone and, especially, the population in general."*
COVID-19 vaccine impact/immunity		

Individual immunity after vaccination	FG1P8	*"Speculating, the number of antibodies that is generated may not be enough to guarantee lasting immunity."*
Group immunity	FG2P1	*"My big concern is that we don't reach that herd immunity that we all need because […] the speed at which people are being vaccinated is very slow, in relation to the propagation speed of the virus."*
Immunity after infection	FG1P4	*"I don't know to what extent I will now have some immunity, given that I have already been exposed to the virus. I would really like to be able to perform/take some serological test or similar."*
Vaccination effects	FG2P3	*"The concern is that we won't get immunity. I'm not worried, honestly, about the issues of adverse reactions, it's really whether vaccination will be enough and that we'll be on this [pandemic] for a while, I'm afraid."*
End of the pandemic	FG2P7	*"At this moment, we have no other solution to get rid of it [the pandemic] other than the vaccine. The vaccine is the hope that we have that, at the end of the tunnel, it will solve it [the pandemic]."*
COVID-19 vaccination-related information		
Missing information	FG2P5	*"People do not know the number of doctors, technicians, researchers, scientists who are behind a vaccine that is about to be approved, and I think that this whole process could be presented to the population a little bit, so that they could understand that it doesn't come out of nowhere."*
Communications through media and social media	FG1P7	*"I think that the media should have a more educational role here, but it never did."*
Information update	FG1P8	*"[…] the way that information arrived and the constant updating of what was true today could be less true tomorrow, which caused some form of anguish."*
Institutional information	FG2P3	*"But I think the DGS has done a good job, at least the website, whenever I consulted it, to clear up any doubts at the beginning, the symptoms, the cases, the evolution. I think they have made a significant effort to invest in the site and the information that is posted there."*
Literacy and language	FG2P3	*"Literacy is very important, so are strategies to bring what is done within the scientific community a little closer together and make it more accessible to the population in all areas."*
COVID-19 vaccination hesitancy/acceptance		
Risk perception	FG1P7	*"Some people still say that it doesn't make sense [to take the vaccine], that they are not part of a risk group and, therefore, they think that even if they contract the disease, they will be asymptomatic or that they will not transmit it."*
Vaccines' effectiveness and safety	FG1P1	*"Regarding safety, there are many vaccines, there is not just one and from what we have already discussed here, I think we can be confident."*
Trust	FG1P8	*"I took both doses of the Pfizer vaccine, I had no adverse effects, no reaction. And I wasn't worried because there was another variable that was at play, which was the prestige of pharmaceutical companies, right? They cannot launch a product on the market that will jeopardize everything they have consolidated over the years."*
Access to information	FG2P5	*"It's really that thing: "now I'm going to see how it happens to others and then I'll decide whether I take it or not". In the small area where I live, I know a lot of people who are in favor of the [national] vaccination plan, but they don't want to take this vaccine and it is certainly due to the lack of information."*
Context	FG2P2	*"I think if this question were asked a month ago, before you started having 300 deaths a day, you would find many more resistant people, i.e. the percentage of vaccine resistant people would be much higher. At the moment, the numbers are an incentive to vaccination."*
